# *Candidozyma auris* Prosthetic Joint Infection: A Case Report and Systematic Case-Based Literature Review of Osteoarticular Infections

**DOI:** 10.3390/pathogens15101057

**Published:** 2026-10-07

**Authors:** Dimitrios Kouroupis, Anastasia-Izampella Papathanasiou, Konstantinos Bourtzinakos, Maria Sentona, Magdalini Kolaza, Kyriaki Vafeidou, Argyro Ftergioti, Symela Gyltidou, Christos Gekas, Charalampos Zarras, Elias Iosifidis, Emmanuel Roilides, Athina Pyrpasopoulou

**Affiliations:** 1 2nd Propedeutic Department of Internal Medicine, Aristotle University School of Medicine, 54124 Thessaloniki, Greece; dimcour841@gmail.com (D.K.); kiriakivf@gmail.com (K.V.); 2 3rd Department of Pediatrics, Aristotle University School of Medicine, 54124 Thessaloniki, Greece; anizelidi@yahoo.gr (A.-I.P.); arofter@gmail.com (A.F.); iosifidish@gmail.com (E.I.); roilides@gmail.com (E.R.); 3 Department of Orthopedics, Hippokration Hospital, 54642 Thessaloniki, Greece; kbourtzinakos@gmail.com (K.B.); sentona.maria@gmail.com (M.S.); gekaschris@yahoo.gr (C.G.); 4 4th Department of Internal Medicine, Aristotle University School of Medicine, 54124 Thessaloniki, Greece; magdakolaza@hotmail.com (M.K.); melina_giltidou@yahoo.gr (S.G.); 5 Microbiology Department, Hippokration Hospital, 54642 Thessaloniki, Greece; zarraschak6@gmail.com; 6 Infectious Diseases Unit, Hippokration Hospital, 54642 Thessaloniki, Greece

**Keywords:** *Candidozyma auris*, osteoarticular infections, systematic case-based literature review

## Abstract

*Candidozyma auris*, recently taxonomically reclassified, represents an emerging multidrug-resistant fungal pathogen that has evolved into a critical nosocomial and global public health threat. This organism exhibits a profound capacity for resilient biofilm formation on biotic and abiotic surfaces, leading to severe systemic infections with highly constrained therapeutic options. This report describes a rare, highly challenging case of a *C. auris*-associated prosthetic joint infection (PJI) in a diabetic patient, representing the 5th analogous documented case (and the 17th osteoarticular infection overall) in the medical literature. The clinical course was characterized by an initial failure to respond to standard echinocandin monotherapy, necessitating the escalation to a synergistic combination regimen. Although temporary microbiological and clinical clearance was achieved, the patient ultimately succumbed to multi-organ failure secondary to a subsequent Gram-negative septic outbreak. Fungal PJIs remain rare clinical entities associated with extreme morbidity and treatment failure. This systematic review emphasizes the crucial need for enhanced infection control strategies and specialized therapeutic protocols as *C. auris* infections continue to rise within everyday clinical environments.

## 1. Introduction

Prosthetic joint infections (PJI) remain among the most devastating complications following total joint arthroplasty, frequently culminating in mechanical failure and the necessity for complex revision surgeries [1,2]. While the vast majority of cases are driven by Gram-positive bacterial strains, especially *Staphylococcus* spp., and Gram-negative pathogens [3], other microorganisms can also be involved, including fungi, particularly *Candida* spp. PJIs due to *Candida* spp. are rare and represent a profound therapeutic challenge due to the historical absence of standardized international guidelines and the resulting high variability in clinical approaches [4]. Currently, the gold standard for treatment centers on two-stage revision surgery; however, success rates remain low and highly variable. Furthermore, the efficacy of antifungal-loaded cement spacers remains controversial [5,6]. Risk factors for *Candida* infection include immunosuppression, systemic disease, and prolonged antibiotic use, which disrupts the host microbiome. Patient colonization and biofilm formation on the skin surface or foreign inserted material usually precede and predispose individuals to invasive infection. These infections primarily occur via contiguous tissue spread or direct inoculation during surgical procedures. The sessile, community-based existence of microorganisms in biofilms is critical for their persistence, the development of clinical infections, and their relapse [7,8,9]. *Candida* spp. can switch from yeast to filamentous growth forms, which more easily invade host tissues. Moreover, virulence factors, such as cellular adherence and the secretion of hydrolytic enzymes, may also strongly influence tissue invasion and PJI development [10]. Modifying these mechanisms and interfering with biofilm assembly represent promising targets for the prevention and management of these infections.

Since its first isolation and identification in 2009, *C. auris* has emerged as a formidable *non-albicans* species, ranking second or third in the frequency of candidemia [11,12]. Its rapid emergence and dissemination have raised profound global concern, prompting its classification as a critical public health threat due to its multidrug-resistant profile and the high mortality rates associated with its invasive infections. Nevertheless, documented fungal osteoarticular infections in general—and fungal PJIs, in particular—remain exceedingly rare, complicating only approximately 1.3% of all PJIs, with Candida species accounting for over 90% of these fungal cases [13]. Epidemiology and etiology vary geographically. To date, fewer than five definitive cases of *C. auris* prosthetic joint infections have been described globally [14].

We present the case of a patient with severe comorbidities and a history of right knee total arthroplasty complicated by *C. auris*—associated PJI, leading to prolonged hospitalization, multiple revisions, and secondary superinfections. Informed consent for publication was obtained postmortem from the patient’s next of kin. This report aims to enhance clinical awareness of both infection prevention measures and management of analogous infections, which are increasingly anticipated in everyday clinical practice.

## 2. Heading

### 2.1. Case Presentation

A 75-year-old woman with a history of diabetes mellitus and heterozygous β-thalassemia was admitted to the Medical Ward of a tertiary hospital from a Rehabilitation Facility in January 2025 due to sepsis. The patient had a surgical history of a right total knee arthroplasty performed in 2015. In November 2024, she sustained a periprosthetic knee fracture that was managed via external fixation, after which she was transferred to the rehabilitation facility. Her subsequent course was complicated by an admission for aspiration pneumonia with secondary *Acinetobacter baumannii* bacteremia. Following a brief return to the rehabilitation center, she developed acute sepsis secondary to a urinary tract infection, necessitating her readmission. During this hospital stay, routine surveillance cultures identified cutaneous colonization with *Candidozyma auris.* The patient was placed on isolation and the infection control measures protocol of the hospital was followed during her hospitalization.

Following recovery, the patient experienced a recurrent episode of pneumonia, necessitating an approximately three-month hospitalization at another hospital where she received prolonged, broad-spectrum antimicrobial therapy. During that admission, her hospital course was further complicated by *Clostridioides difficile* colonization and infection.

In March 2025, the external fixation was converted to intramedullary nailing (Figure 1a). During the postoperative follow-up period, the patient developed a surgical site infection; wound cultures subsequently isolated *Staphylococcus aureus* and *Enterococcus faecalis*. The patient was initially managed with targeted, culture-guided antimicrobial therapy.

On 24 May 2025, the patient was readmitted presenting with localized signs of wound infection and significantly elevated inflammatory markers. At that time, routine surveillance cultures for cutaneous colonization were negative for *Candidozyma auris*. As such, no specific isolation or cohorting measures were taken. However, wound cultures isolated *Proteus mirabilis* and *Pseudomonas aeruginosa*, prompting a targeted adjustment of the antimicrobial regimen. Due to persistent pyrexia, empirical antifungal therapy with anidulafungin was initiated on 1 July.

Due to the ongoing, uncontrolled infection, orthopedic revision surgery was performed. On 9 July, the prosthetic components were removed, and a high-viscosity, radiopaque bone cement spacer loaded with gentamicin (PALACOS^®^ R+G) was inserted. The patient was then transferred back to the medical ward with a negative-pressure wound therapy system (vacuum-assisted closure [VAC]) in place (Figure 1b). Persistent pyrexia after 16 July necessitated a further escalation of the antimicrobial regimen. Subsequent blood cultures isolated *Candida parapsilosis* and *Staphylococcus aureus*; notably, *C. parapsilosis* was also recovered from the tip of the central venous catheter, which had been removed upon the onset of fever.

Follow-up blood cultures demonstrated a polymicrobial bloodstream infection involving *C. parapsilosis*, *Pseudomonas aeruginosa*, and *Staphylococcus haemolyticus*. Antimicrobial therapy was tailored based on susceptibility profiles and included ceftazidime–avibactam, colistimethate sodium, cefiderocol, aztreonam, and fidaxomicin. Antifungal treatment with anidulafungin was maintained until microbiological clearance of the bloodstream infection was achieved, and the patient was subsequently discharged (Table 1).

In September 2025, the patient underwent further surgical wound debridement. On 26 November, revision surgery with reimplantation of a total knee arthroplasty prosthesis was performed (Figure 1c). Prior to the procedure, a joint fluid aspirate was obtained for culture, which yielded negative results. Additionally, five intraoperative tissue culture sets were collected, none of which isolated any pathogens. Gentamicin-enriched bone cement was utilized for the reimplantation. Despite these intraoperative clear cultures, *Enterococcus faecalis* was re-isolated from postoperative wound specimens, resulting in a significantly prolonged hospital stay. Over the following weeks, the patient’s course was complicated by recurrent infectious episodes that required repeated modifications of the antimicrobial regimen.

On 24 December, *C. auris* was isolated from urine cultures. Infection control measures for *C. auris* isolation were reinstituted. Two days later, the same organism was recovered from blood cultures, and two weeks thereafter, it was isolated from wound specimens. Surgical debridement was performed, and the patient was initiated on ampicillin/sulbactam and teicoplanin. Given the patient’s elevated body mass index (BMI: 38 kg/m^2^) and the biofilm-associated nature of the infection, a high-dose anidulafungin regimen (200 mg daily), instead of the approved fixed dose of 100 mg daily, was planned for a 28-day course, following the ID Specialist consultation. She was subsequently discharged to a rehabilitation facility for physical therapy while continuing ampicillin/sulbactam and the high-dose anidulafungin regimen.

Two weeks post-discharge, the patient developed new-onset sepsis and was readmitted. She presented with a *C. auris*-driven PJI, *Pseudomonas aeruginosa* bacteremia (absent in wound cultures), and acute kidney injury (AKI) with an estimated glomerular filtration rate (eGFR) of 17 mL/min/1.73 m^2^. Although colistimethate sodium had been initiated at the rehabilitation center for the septic episode, the AKI was attributed to drug-induced nephrotoxicity rather than hemodynamic instability. Upon readmission, the surgical wound exhibited dehiscence with purulent drainage. *C. auris* was repeatedly isolated from consecutive deep intraoperative tissue cultures through February 2026 (Table 1).

Pathogen identification and antifungal susceptibility testing were performed using the bioMérieux VITEK^®^ 2 system (bioMérieux, Marcy l’Etoile, France), and identification was confirmed via Matrix-Assisted Laser Desorption/Ionization Time-of-Flight Mass Spectrometry (MALDI-TOF MS). Both the index isolate and the subsequent surgical site strains exhibited identical minimal inhibitory concentration (MIC) profiles: amphotericin B (1 mg/L), fluconazole (256 mg/L), voriconazole (0.25 mg/L), isavuconazole (0.03 mg/L), itraconazole (0.03 mg/L), posaconazole (0.015 mg/L), caspofungin (0.06 mg/L), micafungin (0.12 mg/L), and anidulafungin (0.06 mg/L). This phenotypic susceptibility profile remained unchanged throughout the patient’s hospitalization and treatment course. Strains were classified as susceptible or resistant using the European Committee on Antimicrobial Susceptibility Testing (EUCAST) breakpoints.

Given the patient’s clinical status and extensive comorbidities, a second revision surgery was deemed inappropriate. The patient was elderly, diabetic, obese (BMI: 38 kg/m^2^), had undergone multiple major surgeries, and had recently recovered from severe AKI; consequently, she was considered an unfit candidate for further major surgical intervention. Following the resolution of her renal impairment and due to persistent *C. auris* isolation from wound cultures, liposomal amphotericin B (4 mg/kg) was added to the anidulafungin regimen. This combination therapy was maintained for eight weeks until definitive wound culture clearance was achieved. Liposomal amphotericin B was then discontinued, and high-dose anidulafungin monotherapy was maintained. Although the affected knee’s synovial membrane failed to epithelialize, prompting evaluation for plastic synovial reconstruction, the procedure was deferred due to the recent localized infection.

The patient’s clinical course was further complicated by a new episode of sepsis due to *Klebsiella pneumoniae* with associated PJI, culminating in the development of multi-organ failure that required immediate transfer to the Intensive Care Unit (ICU). Notably, *C. auris* was again isolated from wound cultures during this acute episode. Despite aggressive intervention, the patient succumbed to multi-organ failure and passed away on 17 April, 12 days following her ICU admission.

### 2.2. Literature Search

The objective of this systematic case-based literature review was to analyze published cases of *C. auris*-associated osteoarticular infections. The literature search was conducted in MEDLINE/PubMed, Scopus, Web of Science, Google Scholar and Directory of Open Access Journals (DOAJ). For the search, Medical Subject Headings (MeSH) and free-text terms were combined using Boolean operators (AND/OR). The search strategy included terms related to case report (presentation, series), *C. auris* (*Candida auris* or *Candidozyma auris*), and osteoarticular involvement (bone, joint, septic arthritis, skeletal, infection, osteomyelitis, spondylodiscitis, prosthetic joint, osteoarticular infection). The search string was applied on 20 August 2026. All cases with microbiologically proven *C. auris* osteoarticular infection were included. Cases and case series involving prosthetic joint infections were summarized in Table 2; cases involving native bone/ joint infections were summarized in Supplementary Data Table S1, respectively.

No restriction was set on publication date; all eligible case reports and case series studies published up to the final search date were considered for inclusion. The components of the search within the identified relevant literature were defined as follows: Population: patients of both genders and all age groups. Intervention/Exposure: proven *C. auris* native and prosthetic osteoarticular infection, Year of publication and author, Patient gender, age (years), Site/Type of Infection, History/Risk factors, *C. auris* clade, Primary source, Treatment/Duration and Outcome. Wherever unavailable, data were filled in as “Unknown”.

The search identified a total of 16 previously documented cases of proven *C. auris* osteoarticular involvement across 15 separate peer-reviewed publications [15,16,17,18,19,20,21,22,23,24,25,26,27,28,29]. Twelve cases involved osteoarticular infections of native bones and joints causally associated with *C. auris* [15,16,17,18,19,20,21,22,23,24,25] and four (4) cases involved infection of prosthetic material (arthroplasty) [26,27,28,29]. In one of the latter four patients, the cement spacer was infected after the removal of the prosthesis during revision surgery. Demographics of the patients (gender, age), patients’ history, the site of the infection and/or type of prosthesis, phylogenetic clade of the strain (if known), primary source of the pathogen (in cases in which information was available), type and duration of treatment, and patients’ outcome are summarized in Table 2 (prosthetic joints) and Supplementary Data Table S1 (native joints). Eradication of the infection was recorded in 5/15 (33%) patients (in 4 native osteoarticular infections and in 1 prosthetic one). In the remaining 10 cases, the patient had either died (all-cause death) or the infection was still ongoing. Prosthetic joint infection treatment appears to have smaller success rates (25% vs. 40%) and higher rates of relapse (60%). Still, these numbers need to be addressed with caution, as there is very little clinical experience with such infections.

**Table 2:** Systematic extraction of documented *C. auris* prosthetic joint Infections and their characteristics; the 3 first cases refer to PJIs, and the last case refers to contamination of the cement spacer which was placed in the course of revision surgery (*TKA: total knee arthroplasty*).

Case ID and Source	Demographics and Site	Clinical Risk Factors	Clade	Antifungal Treatment Regimen	Clinical Outcome
**Case 1** **Current Case** **(2026)**	Female, 75 Yrs Right Knee (TKA)	Diabetes Mellitus, Obesity (BMI 38), Broad Antibiotic Exposure	Unknown	High-dose Anidulafungin (200 mg/d) + Liposomal Amphotericin B (4 mg/kg/d) for 2 months.	Local Clearance; Patient expired due to secondary K. pneumoniae sepsis.
Case 2 Spruijtenburg (2025) [26]	Unknown, >50 Yrs, Total Hip Joint	Prior prolonged broad-spectrum antimicrobial therapies	Clade I	Complete prosthesis removal; Micafungin monotherapy for 3 to 6 months.	Relapse at 3 months; Required subsequent debridement and prolonged treatment.
Case 3 Patel M. (2025) [27]	Male, 60 Yrs Right Knee (TKA)	Obesity, Hypertension, Hypothyroidism, Multiple prior revisions	Unknown	Retention of hardware; joint irrigation, debridement, polyethylene exchange + Micafungin (6 weeks).	Successful Resolution; Sustained clinical and lab clearance at 3-month follow-up.
Case 4 Mohan V. (2020) [28]	Male, 70 Yrs Left Knee (TKA)	Diabetes Mellitus, prior extensive antimicrobials, previous TKA failure	Unknown	Staged surgical revision and debridement; High-dose Caspofungin + local Amphotericin B (2 months).	Initial Resolution; Recurrent infection documented 1.5 years later requiring arthrodesis.
Case 5 Roberts S.C. (2019) [29]	Female, 78 Yrs Left Ankle Spacer	Multiple prior ankle surgical interventions, broad antimicrobial access	Unknown	Spacer excision and radical debridement; new cement spacer with Amphotericin B + Micafungin (2 weeks), then oral Fluconazole.	Clinical Improvement; Stable and infection-free at the time of publication of the journal report.

## 3. Discussion

The yeast *C. auris* (formerly *Candida auris*) has rapidly emerged as an important causative agent of invasive infections within healthcare settings [30,31]. Due to its ability to colonize human skin and its prolonged survival in the hospital environment and on medical devices, this pathogen can cause persistent clonal outbreaks and invasive infections with mortality rates averaging ∼30%, though figures vary considerably by geographical region and patient cohort [30,32]. Because *C. auris* exhibits near-universal resistance to azoles (ranging from 94% to 96%, particularly within clades I and III) [33,34,35], echinocandins have become established as the first-line therapy associated with a distinct survival benefit [36]. However, prolonged or repeated exposure to echinocandins can induce clinical resistance. This resistance mechanism is primarily driven by mutations in the drug-target gene *fks1*, which encodes the catalytic subunit of β-(1,3)-D-glucan synthase. Consequently, as the epidemiological incidence of *C. auris* rises and inevitably drives an increase in echinocandin utilization, the emergence of echinocandin resistance is being documented with growing frequency [37].

*Candidozyma auris* exhibits an extraordinary phenotypic capacity to switch from single-celled yeast forms into highly aggregative, multicellular communities embedded within a dense, self-produced extracellular polysaccharide matrix [38,39]. This architectural barrier severely impedes the physical penetration of most conventional antifungal agents. Genetic modifications conferring adaptive advantages include mutations in genes required for *Candida* filamentous growth, chitin synthase-encoding genes, and other virulence-associated factors [40,41]. The clinical presentation of *C. auris* is typically nonspecific and emulates other types of systemic fungal infections [42]. The vast majority of documented cases occur in adults, with critically ill individuals exhibiting the highest prevalence rates. This increased vulnerability is primarily driven by the concomitant presence of established risk factors, such as indwelling urinary or central venous catheters, underlying malignancy, renal dysfunction, and neutropenia. Epidemiological studies have demonstrated that patients receiving prolonged courses of broad-spectrum antibiotics face an elevated risk of acquiring resistant fungal infections, which frequently originate from intestinal or cutaneous colonization. In these cohorts, pre-existing *C. auris* colonization is the primary driver of subsequent invasive disease—a clinical trajectory that directly mirrors the course observed in our patient [35].

Failure to eradicate the systemic fungal infection is associated with clinically refractory candidemia and significant morbidity [43] and is largely attributed to the prolonged persistence of the pathogen on both biotic and abiotic surfaces, as observed in our patient. Our patient was managed with a prolonged, combination antifungal regimen. Despite achieving multiple, consecutively negative fungal cultures from the affected surgical site, the pathogen critically re-emerged in cultures three months later. The efficacy of currently available antifungal agents against *Candida* biofilms has been extensively evaluated in the literature, revealing that the impact of these drugs on biofilm formation and structural integrity is highly complex. For instance, fluconazole can inhibit biofilm assembly if it exhibits intrinsic activity against the implicated fungal strain; however, it remains largely ineffective against pre-formed structures, despite its ability to readily penetrate the dense biofilm matrix [44,45]. This clinical limitation is primarily attributed to the development of markedly elevated minimal inhibitory concentration (MIC) values among the fungal cells residing within the biofilm [46,47]. Conversely, in experimental in vitro diffusion cell bioassay systems, amphotericin B demonstrated the lowest penetration through fungal biofilms; yet, it induced the most profound structural damage to the superficial cells of the biofilm matrix compared to its comparators, fluconazole and flucytosine [48]. Despite these distinct matrix-penetration challenges, echinocandins remain the recommended first-line agents in the therapeutic arsenal against systemic *Candida auris* infections [49,50,51].

Treating biofilm-associated *Candida* infections remains a formidable clinical challenge; consequently, international guidelines strongly advocate for the immediate removal of central venous catheters (CVCs) in cases of microbiologically documented candidemia [52,53]. However, because our patient was elderly, obese, and presented with severe comorbidities—having already undergone multiple revision surgeries of the right knee joint—further prosthetic component removal was deemed unsafe. Instead, antifungal agents with demonstrated efficacy against biofilm structures were initiated. The *C. auris*-associated PJI was initially managed with an escalated dose of anidulafungin, rather than the standard recommended dosing regimen, to promote rapid sterilization of the prosthetic components. However, the persistent failure to achieve microbiological clearance from joint fluid aspirates after more than a month of monotherapy (spanning from her discharge to her readmission with sepsis) necessitated treatment escalation via the addition of amphotericin B to the regimen. Combination antifungal therapy is typically reserved as salvage treatment in analogous complex cases, yielding highly variable clinical outcomes and a pronounced risk of secondary adverse effects [15,16]. In addition to systemic therapy, local peri-articular irrigation with an amphotericin B solution, diluted in sterile water for injection, was intraoperatively applied to the peri-articular space during negative-pressure wound therapy (vacuum-assisted closure [VAC]) dressing changes. Local amphotericin B irrigation has been documented in isolated case reports as an empirical salvage strategy with inconsistent outcomes, primarily within the context of *Aspergillus*-associated infections [54]. Notably, its utilization in native joints is strictly limited by significant chondrotoxicity [55]. In a recent metanalysis including 385 *Candida*-associated PJIs from 110 publications, first-line therapy with amphotericin B and complete hardware removal were the only two parameters associated with favorable clinical outcome [56]. After 2 months of combined treatment and the establishment of mild renal impairment (47ml/min, estimated GFR—Cockcroft Gault formula) amphotericin B was discontinued. Sterilization of the wound culture was achieved (Table 1). Infection, however, relapsed 2 months later together with new-onset bacterial superinfection (Table 1). The patient was maintained on high-dose anidulafungin until her death. Had she survived, antifungal management would have required an indefinite course of long-term suppressive therapy, presenting highly constrained and questionable therapeutic options.

Including the present report, a total of 17 cases of *C. auris*-associated osteoarticular infections have been documented in the literature, with 5 specifically involving prosthetic joints (Table 2 and Supplementary Data Table S1). Given the limited number of cases and the heterogeneity thereof, no safe assumptions can be drawn regarding the appropriateness of management. Successful resolution was achieved in only 5 cases overall (comprising 5 out of 12 native bone/joint infections and 1 out of 5 prosthetic joint infections); in the remaining cases, the patient either succumbed to concurrent medical complications, experienced infectious relapse, or remained under active treatment [15,16,17,18,19,20,21,22,23,24,25,26,27,28,29]. To safely extrapolate conclusions regarding the success of management, longer follow-up periods are mandatory. In most cases, treatment was extended temporally beyond the formulated guidelines. Among these cases, infection resolution—particularly within the context of PJIs—was exclusively documented following the complete removal of the prosthetic components. Although our patient initially achieved multiple consecutive negative wound cultures following the initiation of combination antifungal therapy, indicating an apparent clearance of the fungal PJI, the localized *C. auris* infection ultimately relapsed. This recurrence coincided with a new, and ultimately fatal, episode of sepsis driven by a secondary bacterial joint infection. Critically, it must be emphasized that our patient had been continuously hospitalized over the preceding two years, experiencing multiple septic episodes that necessitated the prolonged administration of various broad-spectrum antimicrobial regimens.

Since its first isolation in Japan in 2009, *C. auris* has spread worldwide, with documented cases reported in more than 47 countries. In certain regions, the epidemiological situation has escalated to endemic status [57,58]. Surveillance data from Greece indicate an exponential increase in cases over the past two years, categorizing the country under epidemiological spread grade 4 (on a scale of 0–5) [59]. Given its epidemiological significance, the persistence of its colonization, and the severe burden its associated systemic infections impose on clinical outcomes, patient quality of life, and healthcare economics, the implementation of rigorous infection prevention, control, and surveillance measures is of paramount importance. These interventions comprehensively include educating operating room, inpatient, and outpatient staff regarding the organism and mandated containment measures. In the surgical setting, the protocol dictates the use of surgical space suits and shoe covers by all operating room personnel, alongside the decontamination of operating rooms and perioperative care units using bleach and ultraviolet (UV) light technology. On inpatient wards, strict contact precautions—including the mandatory use of gowns and gloves—must be enforced, ideally supplemented by 1:1 nursing care. Furthermore, daily patient room cleanings should be executed utilizing bleach-based solutions, and the sharing of medical equipment must be strictly limited, ensuring devices are either disposed of when feasible or meticulously decontaminated [26].

There are limitations in both the case report and the case-based literature review. With regard to the case report, the isolates obtained from colonization and from the prosthetic infection were not tested to determine whether they belong to the same or a different strain. With regard to the literature review, we specifically mention the very small number of reported *C. auris*-associated osteoarticular infections, the heterogeneity of surgical approaches and antifungal regimens, the lack of standardized treatment strategies, the limited duration of follow-up in some published cases, and the consequent difficulty in drawing conclusions regarding the efficacy of specific antifungal combinations or surgical approaches. These limitations are inherent to rare diseases and the review of the few case reports published in the literature.

## 4. Conclusions

Prosthetic joint infections (PJIs) represent severe, debilitating, and therapeutically challenging complications, particularly when they manifest in elderly patients with multiple comorbidities. As one of the primary drivers of total arthroplasty failure, PJIs impose an immense clinical burden. Among these, *Candida*-associated PJIs are exceedingly rare, and the optimal strategies for their surgical management and companion antifungal therapy remain poorly defined. Eradication frequently necessitates complex revision surgeries and protracted hospitalizations, largely due to the requirements of prolonged intravenous antifungal administration. The sparse number of *Candidozyma auris*-associated joint infections documented globally to date underscores the rarity of this clinical entity and highlights the critical need for additional clinical evidence to establish standardized management protocols. The present case contributes to a growing body of evidence indicating that *C. auris* must be considered a potential, highly resilient etiology of PJI, especially in patients with extensive healthcare exposure and established risk factors. Because this multidrug-resistant pathogen is anticipated to cause growing concern within the global medical community, the timely implementation of rigorous infection prevention, control, and surveillance measures remains a high priority.

## Figures and Tables

**Figure 1:**
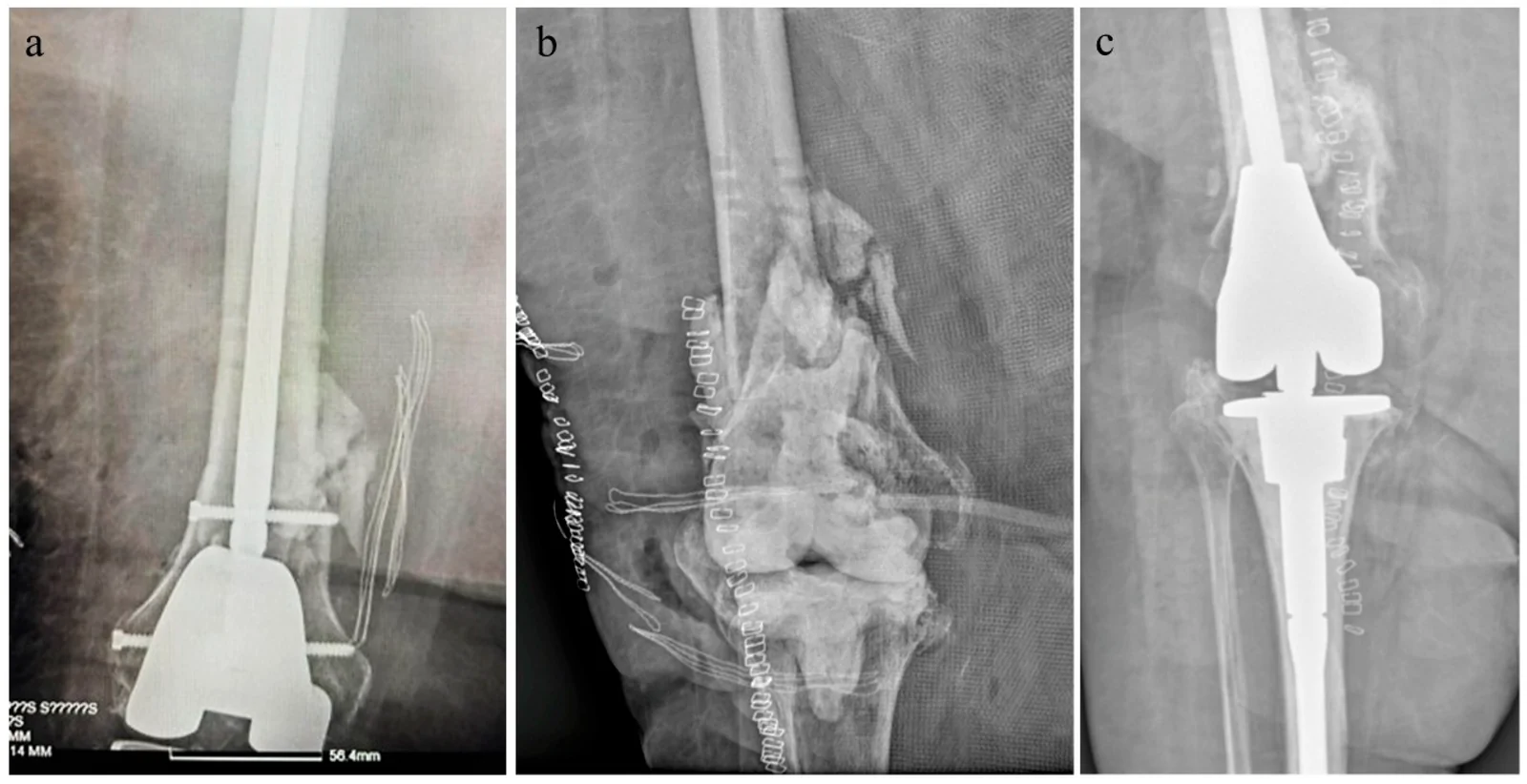
X-Ray image of the infected right knee joint arthroplasty before (**a**) and after (**b**) the removal of the prosthetic material. (**b**) Cement has been placed in the joint and negative-pressure wound therapy (VAC) has been applied. (**c**) Radiology after revision surgery with reimplantation of a total knee arthroplasty prosthesis.

**Table 1:** Microbiological cultures from clinical samples of the patient, listed chronologically since the patient’s first admission in January 2025. Urine and skin cultures growing fungi were considered colonization; all other positive cultures represent true bacterial/fungal infections. (*Neg: negative; CVC: central venous catheter; BAL: Bronchoalveolar; arrow indicates ongoing continuous admission).*

Date	Blood Culture Aerobic	Blood Culture Anaerobic	CVC Tip Culture	Wound Culture	BAL Culture	Urine Culture	Skin Colonization for *C. auris*
First Admission (*Acinetobacter baumanii* Associated Bacteremia)							
24 Decmber	*A. baumanii*						
Second admission (Urosepsis)							
13 January 2025							*C. auris*
Third admission (Sepsis due to infected knee joint prosthesis)							
23 May 2025						Neg (−)	Neg (−)
26 May 2025				*S. aureus*/*E. faecalis*/*Pantoea* spp./CoNS			
29 May 2025	Neg (−)	CoNS		*E. faecalis*/CoNS/*A. baumanii*			
9 July 2025	*S. sciuri*	*S. sciuri*	*P. mirabilis*/*K. pneumoniae*			*C. orthopsilosis*	
Transfer to the Orthopedics Department—Surgical removal of infected prosthetic knee joint							
18 July 2025	Neg (−)	Neg (−)					
21 July 2025	*S.haemolyticus*/*C. parapsilosis*	Neg (−)	*C. parapsilosis*				
28 July 2025	*S.haemolyticus*/*C. parapsilosis*/*P. aeruginosa*	Neg (−)	*P. aeruginosa*				
30 July 2025	*P. aeruginosa*	Neg (−)					
5 August 2025	*P. aeruginosa*	Neg (−)					
7 August 2025	*S. hominis*	*S. hominis*					
10 August 2025	*C. parapsilosis*	Neg (−)					
14 August 2025	*C. parapsilosis*						
18 August 2025	Neg (−)						
14 September 2025	Neg (−)	Neg (−)					Neg (−)
Fourth admission (Admission for reimplantation of a prosthetic knee joint); days are numbered from reimplantation surgery							
Day 1						*P. mirabilis*	
Day 5				*E. faecalis*		*P. aeruginosa*	
Day 23				*Neisseria sicca* group		*C. albicans*	
Day 29	*A. baumanii*	Neg (−)		Neg (−)		*C. auris*	
Day 31	*C. auris*	Neg (−)		Neg (−)		*C. auris*	
Day 37	*P. aeruginosa*	*P. aeruginosa*		Neg (−)			
Day 46			*P. aeruginosa*/*P. mirabilis*	*C. auris*		*P. aeruginosa*/*C. auris*	
Day 50				*C. auris*		*C. albicans*	
Readmission from the Rehabilitation Center due to sepsis							
Day 77	*P. aeruginosa*	Neg (−)		*C. auris*		*C. auris*	
Day 82	*A. baumanii*	*A. baumanii/P aeuginosa*/CoNS.		*C. auris*		*C. auris*	
Day 86	Neg (−)	Neg (−)					
Day 97	*C. parapsilosis*	*S. epidermidis*					
Day 114	*S. haemolyticus*	*S. haemolyticus*		Neg (−)		*P. aeruginosa*/*C. auris*	
Day 116	Neg (−)	Neg (−)					
Day 131				*K. pneumoniae*		*C. auris*	
Day 139	Neg (−)	Neg (−)		*P. aeruginosa*/*C. auris*			
Day 143	Neg (−)	Neg (−)			*P. aeruginosa*/*C. auris*		*C. auris*
Day 148	Neg (−)	Neg (−)			*P. aeruginosa*/*C. auris*		*C. auris*

## Data Availability

The original contributions presented in this study are included in the article/Supplementary Material. Further inquiries can be directed to the corresponding author.

## Supplementary Materials

The following supporting information can be downloaded at: https://www.mdpi.com/article/10.3390/pathogens15101057/s1, Table S1: Synopsis of reported *Candidozyma auris*-associated cases of native bone and joint infections and their characteristics (*yrs: years; ESRD: end-stage renal disease; DM: diabetes mellitus; ICU: intensive care unit; COPD: chronic obstructive pulmonary disease; mos:months*).

## Abbreviations

The following abbreviations are used in this manuscript:

PJI	Prosthetic Joint Infection
VAC	Vacuum Assisted Closure
BMI	Body Mass Index
AKI	Acute Kidney Injury
eGFR	estimated Glomerular Filtration Rate
MALDI-TOF MS	(Matrix-Assisted Laser Desorption/Ionization Time-of-Flight Mass Spectrometry)
MIC	Minimal Inhibitory Concentration
EUCAST	European Committee on Antimicrobial Susceptibility Testing
ICU	Intensive Care Unit
DOAJ	Directory of Open Access Journals
MESH	Medical Subject Headings
vs.	versus
CVC	Central Venous Catheter
BAL	Bronchoalveolar Lavage
Yrs	years
ESRD	End-Stage Renal Disease
DM	Diabetes Mellitus
COPD	Chronic Obstructive Pulmonary Disease
UV	Ultraviolet
