# Supplementary material for: Candidozyma auris Prosthetic Joint Infection: A Case Report and Systematic Case-Based Literature Review of Osteoarticular Infections
Source: Pathogens. 2026 Oct 7;15(10):1057. doi: 10.3390/pathogens15101057 (PMC13708079; doi:10.3390/pathogens15101057)
Supplement: Supplementary file 1 [file pathogens-15-01057-s001.zip › pathogens-4579539-supplementary.pdf]

Supplementary Table S1. Synopsis of reported *Candidozyma auris*-associated cases of native bone and joint infections and their characteristics (*yrs: years; ESRD: end-stage renal disease; DM: diabetes mellitus; ICU: intensive care unit; COPD: chronic obstructive pulmonary disease; mos:months*)

no #	Publication year, first author	Gender, age(yrs)	Site/Type of Infections	History/Risk factors	Clade	Primary source	Treatment/ Duration	Outcome
**1**	**2026,** Vinith M	Female, 27	Right distal femur osteomyelitis	Previous road traffic accident with closed injury	Unknown	Unknown	Surgical debridement oral voriconazole 6 mo	Resolution (clinical and radiological)
**2**	**2026,** Rodriguez L	Male, 72	Left hip septic arthritis/ Thoracic spine Osteomyelitis-discitis	Guillain-Barre syndrome, quadriplegia, extensive healthcare exposure	Unknown	Unknown	Source control Amphotericin B, Micafungin/ 12 mo (ongoing)	Persistent infection at 9 months, treatment ongoing
**3**	**2025,,** Jenkins	Male 70	Vertebral osteomyelitis	Recurrent catheter associated *C. auris* bloodstream infection	Unknown	Bloodstream infection	Liposomal amphotericin B, micafungin / 7 weeks change to rezafungin because of L-Amb toxicity / 5 mo	Resolution (clinical and radiological)
**4**	**2025,** Sambas R	Male, 65	Spondylodiscitis	Diabetes Melitus, Hypertension, adrenal insufficiency, ESRD/hemodialysis, permanent catheter, vascular disease, prior antimicrobial exposure	Unknown	Unknown	Caspofungin, Liposomal amphotericin B / 1.32 mo until subsequent death	Death
**5**	**2025,** Güler Ö	Male, 41	Vertebral osteoarticular infection	Multiple trauma, surgery for lumbar vertebrae, insulin-dependent DM	Unknown	Unknown	Caspofungin/ 49 days	Infection considered eradicated, clinically and radiologically incompletely resolved
**6**	**2024,** Langourani-Kosteletou P	Male, 73	Spondylodiscitis	Multilevel kyphoplasty/vertebroplasty, diabetes mellitus, myocardial infarction/ permanent pacemaker/ prior exposure to	Unknown	Probable Colonization from	Debridement, posterior decompression and stabilization Micafungin / 12 mo (ongoing)	Clinical improvement at 9 months follow up, treatment ongoing

				antibiotics/ corticosteroids/ICU stay		ICU		
**7**	**2020,** Fernandez-Chagüendo CM	Male, 48	Right femoral osteomyelitis	Previous femoral head fracture/Paraplegia/ neurogenic bladder/ prior antimicrobial exposure	Unknown	Unknown	Debridement, source control/ antifungal therapy not reported	Not reported
**8**	**2020,** Supreeth S	Male, 50	Spondylodiscitis	Sickle cell disease, diabetes mellitus, multiple previous surgeries	Unknown	Unknown	Debridement, posterior decompression and stabilization Caspofungin / 2 mo	Resolution (clinical and radiological)
**9**	**2019,** Heath CH	Male, 65	Sternal osteomyelitis	Severe COPD, pulmonary hypertension, ischemic heart disease, and chronic kidney impairment, previous ICU stay	III	Colonization from ICU	Surgical debridement Posaconazole pos / 3 mo until subsequent death	Death due to cardiorespiratory failure
**10a**	**2018,** Ruiz-Gaitan	Male, 66	Spondylodiscitis	Abdominal surgery	Unknown	Bloodstream infection	Anidulafungin, liposomal amphotericin B followed by Anidulafungin, Posaconazole / 6 mo	Not reported
**10b**	**2018,** Ruiz-Gaitan	Male, 42	Spondylodiscitis	Polytrauma	Unknown	Bloodstream infection	Anidulafungin followed by posaconazole/ 9 mo	Not reported
**11**	**2017,** Choi HI	Male, 54	Bilateral otomastoiditis	Pontine hemorrhage 3 months previously	Unknown	Unknown	Surgical debridement Fluconazole iv followed by fluconazole pos / 143 days oral therapy	Resolution (clinical and radiological)
